# Plasmonic Hybrid Heterostructure Based on Reduced Graphene Oxide-Gold Nanostars Composite for Sensitive SERS Sensing

**DOI:** 10.1177/00037028251344628

**Published:** 2025-08-06

**Authors:** Supriya Atta, Tamer Sharaf, Tuan Vo-Dinh

**Affiliations:** a Fitzpatrick Institute for Photonics, Ain Shams University, Cairo, Egypt; b Department of Biomedical Engineering, Ain Shams University, Cairo, Egypt; c Department of Physics, Ain Shams University, Cairo, Egypt; d Department of Chemistry, Duke University, Durham, NC 27708, USA

**Keywords:** Gold nanostars, graphene oxide, SERS, sensing, pesticides, thiram

## Abstract

In this study, we have developed a plasmonic hybrid heterostructure integrating two elements: (1) two-dimensional (2D) reduced graphene oxide-gold nanostars composite (rGO-GNS), and (2) gold nanostars (GNS) substrate. By harnessing the unique plasmonic properties of rGO in chemical enhancement and that of GNS in electromagnetic enhancement, the hybrid heterostructure offers synergistic enhancement effects that enable ultra-low sensitivity and accurate identification and analysis of trace quantities of target substances. It is noteworthy that the high-density hotspots generated by strong plasmonic coupling of rGO-GNS and GNS results in ultra-high SERS enhancement compared to individual substrate either GNS or rGO-GNS substrate. Moreover, the uniformity and reproducibility of the GNS@rGO-GNS substrate were studied by using thiophenol (TP) as a model analyte, which indicates that the SERS sensor exhibited superior signal reproducibility with an RSD value 5 % and long-term stability with a minimal signal loss after 30 days. To demonstrate a potential application of our SERS substrate, SERS detection of pesticide-thiram in river water was realized with a LOD up to 50 pM, showing the potential for new opportunities for efficient chemical and biological sensing applications.

## Introduction

Surface-enhanced Raman scattering (SERS) has emerged as a versatile and ultrasensitive analytical method for various analytes detection in different fields such as food safety^1–5^, environmental monitoring^6, 7^, biomedical sensing^8–12^, and many more^13–15^. Our laboratory first introduced the use of SERS as an analytical technique for trace organic analysis,^16^and has subsequently developed this technique for a wide variety of sensing applications^17–20^. Compared to other traditional analytical methods such as such as high-performance liquid chromatography (HPLC), gas chromatography (GC), and GC-MS, the main advantage of SERS is that it can directly provide unique fingerprint information of the analytes in complex real-world matrices without any sample repurification^21–24^. The enormous signal enhancement of the SERS effect is attributed to two main processes, electromagnetic enhancement, and chemical enhancement^25–27^. Whereas the chemical enhancement is associated with charge transfer between the target molecules and SERS substrates, the electromagnetic enhancement is attributed the electromagnetic field confinement effect generated by the excitation of localized surface plasmon resonance (LSPR) in noble metal nanostructures^28–30^. This effect dramatically enhances the Raman signals of the target molecules in close proximity to the plasmonic nanostructures, allowing for detection sensitivity down to single molecule level^31^. Anisotropic nanostructures such as nanorods, nanocubes, nanostars, nanotriangle plates, and nanoflowers are particularly attractive as SERS substrates due to their inherent ‘built-in’ hot-spots, which contribute to high SERS enhancement capabilities without the need for complex manipulation^32–36^.

Recently, hybrid multi-plasmonic metal nanoarray composites have attracted attention for achieving ultra-high SERS sensitivity^37^. In this context, the combination of plasmonic nanostructure and graphene or graphene derivatives such as reduced graphene oxide (rGO) as SERS platform has attracted considerable interest due to their unique plasmonic optical properties which makes it possible to get close contact with analyte molecules^38^. rGO also has several advantages over other semiconductor materials. Firstly, rGO is a derivative of graphene, a single layer of carbon atoms arranged in a two-dimensional honeycomb lattice, the large surface area of graphene and rGO provides ample sites for the adsorption of organic analyte molecules such as pesticides, increasing the chances of interaction with the plasmonic nanoparticles^39^. Secondly, the π-π interactions between the aromatic rings of graphene or rGO and the aromatic analyte molecules can enhance the charge transfer process, leading to chemical enhancement of the Raman signals^40^. Additionally, the strong electrostatic interactions between the negatively charged rGO and the positively charged pesticide molecules can facilitate their adsorption onto the graphene surface, further enhancing the detection sensitivity. Moreover, the presence of rGO can induce quenching of fluorescence signals of analyte molecules in the vicinity of graphene or rGO nanosheets^41^. Overall, the combination of plasmonic noble-metallic nanostructures with graphene or rGO provides a promising platform for sensitive SERS detection of pesticides. This approach takes advantage of the unique properties of graphene and its derivatives, as well as the amplification capabilities of plasmonic nanoparticles, to achieve both high sensitivity and molecular specificity in pesticide detection. Therefore, the combination of plasmonic nanoparticles with graphene or reduced graphene oxide (rGO) offers several advantages for sensitive SERS detection of pesticides^42^. A notable development is the utilization of a SERS system consisting of a composite film composed of Ag-nanocubes, rGO, Au-nanoparticles on a hydrophobic surface^42^. Nevertheless, the conventional rGO-gold nanoparticle SERS substrates can generate only a few hotspots as they are mainly 2D array. Therefore, the combination of multi-dimensional rGO-gold nanoparticles can offer multiple coupling, which can enhance the SERS signal of analytes. Thus, this is the focus of our study to develop a substrate with multiple coupling systems that combine rGO-anisotropic nanostructures in a 3D array.

For instance, among different size and shape of gold nanoparticle systems, the anisotropic nanostructure gold nanostars has gained significant momentum in the realm of SERS enhancement owing to their distinct optical properties producing a stronger LSPR due to the lightning rod effect, which makes the SERS effect stronger than that of other nanoparticle systems^43–47^. The decoration of rGO with GNS involves the attachment of these nanostars onto the surface of graphene oxide sheets. The resulting composite material, rGO decorated on GNS, combines the unique properties of both rGO and GNS^48^. rGO provides excellent mechanical strength, high surface area, and good dispersibility, while GNS contributes to their plasmonic properties and unique morphology. This composite material has the potential for a wide range of applications, including biosensing, catalysis, photonics, and nanoelectronics^49^.

Herein, we proposed a novel SERS platform based on gold nanostars-reduced graphene oxide (rGO-GNS) nanocomposite decorated on GNS substrate to realize multiple plasmonic coupling in the hybrid GNS@rGO-GNS nanostructure to achieve the ultrasensitive label-free SERS detection of a pesticide thiram. rGO-GNS was first synthesized with highly spiked morphology of GNS, then the rGO-GNS nanocomposite was embedded on the surface of the GNS substrate. The SERS enhancing capabilities of GNS, rGO-GNS and GNS@rGO-GNS were compared by using a model analyte TP, which shows that the hybrid nanostructure GNS@rGO-GNS has the highest SERS sensitivity. Furthermore, we applied our SERS substrate for the detection of a pesticide-thiram in river water, where we achieve a LOD of up to 50 pM. Overall, our substrate has the potential for applications in environmental monitoring and food safety.

### Experimental section

#### Materials and Characterization

Chloroauric acid (HAuCl_4_), L-ascorbic acid, silver nitrate (AgNO_3_, 99.8%) hydrochloric acid (HCl), trisodium citrate (Na_3_C_6_H_5_O_7_), and thiram were purchased from Sigma-Aldrich. Milli-Q deionized (DI) water was used throughout the experiment. Graphene oxide stock solution (4 mg/mL) was purchased from Graphenea, San Sebastian, Spain. The TEM images of GNS and rGO-GNS were acquired using FEI Tecnai G^2^ Twin TEM system. The GNS and GNS@rGO-GNS substrate were studied by SEM (FEI Verios 460 L). UV-vis spectra were recorded using a FLUOstar Omega microplate reader.

#### Raman measurements

Raman measurements were performed by using a laboratory build portable Raman instrument having a 785-nm laser source (Rigaku Xantus TM-1 handheld Raman device), a fiber optic probe (InPhotonics RamanProbe), a spectrometer (Princeton Instruments Acton LS 785), and a CCD camera (Princeton Instruments PIXIS: 100BR_eXcelon). The laser power of the Rigaku Xantus TM-1 was set at 200 mW, and exposure time was set at 1 sec. A sample volume of 10 μL was used to obtain the Raman signal.

#### Synthesis of GNS

GNS were synthesized by following our previously reported method ^11^. Briefly, 200 μL of 1 N HCl was added to a solution containing 50 mL of 1 mM HAuCl_4_ and 1 mL of 20 nm gold seeds solution. After that, we added a solution of 2 mL of 3 mM AgNO_3_ and 1 mL of 100 mM ascorbic acid to the solution and the solution was stirred for 2 minutes which was used further for substrate preparation.

#### Synthesis of Reduced Graphene Oxide-GNS (rGO-GNS)

rGO-GNS was synthesized by a modified reported method^50^. Briefly, rGO-goldnanoparticles first synthesized, where the mixture of 1 mL graphene oxide aqueous solution (4 mg/mL), 49 mL Milli-Q water and HAuCl_4_ solution (500 μL, 25 mM) was heated at 90 °C for 15 min and then 500 μL of sodium citrate (1.0 M) was added to the solution mixture. The solution mixture was stirred for 1 hour and used for further rGO-GNS synthesis. rGO-GNS was synthesized by a modified seed-mediated growth method.^50^ Briefly, 200 μL of 1 N HCl was added to a solution containing 50 mL of 1 mM HAuCl_4_ and 500 μL of rGO-goldnanoparticles solution. After that, we added a solution of 2 mL of 3 mM AgNO_3_ and 1 mL of 100 mM ascorbic acid to the solution and the solution was stirred for 2 minutes which was used further for substrate preparation.

#### Preparation of GNS@rGO-GNS substrate

APTES-functionalized Si-wafer substrate was prepared according to the literature procedures^51^. The Si-wafer substrates were washed with Aqua Regia (HCl: HNO_3_ in a 3:1 ratio by volume) and rinsed with Milli-Q water three times. The Si-wafer substrates were further cleaned in ethanol with sonication three times and dried at 100 °C for 1 h in an oven. The cleaned Si-wafer substrates were then dipped in a 1% (v/v) ethanol solution of APTES in ethanol at 70 °C for 2 h. After that, the substrates were rinsed three times in ethanol with sonication to remove excess APTES and dried for 2 h at 100 °C in an oven. Then, the Si-wafer substrates were vertically immersed overnight into the GNS solution and washed with ultrapure water gently once and dried. The solution of as-synthesized rGO-GNS was added to the GNS substrate for a short period of time (15 minutes) and washed with ultrapure water gently once and dried.

## RESULTS AND DISCUSSION

It is well-known that the plasmonic rGO-GNS composite can generate a large number of inherent “hotspots” resulting in remarkable enhancement in the overall SERS signal response in comparison with gold nanoparticles because of the synergetic effect of both the electromagnetic enhancement induced by plasmonic nanoparticles and chemical enhancement induced by rGO^50, 52, 53^. Therefore, we believe that the fabrication of rGO-GNS assembly over GNS can generate a higher number of inherent “hotspots” resulting in remarkable enhancement in the overall SERS signal response.

### GNS@rGO-GNS Substrate Preparation and Characterization

**Figure 1.**
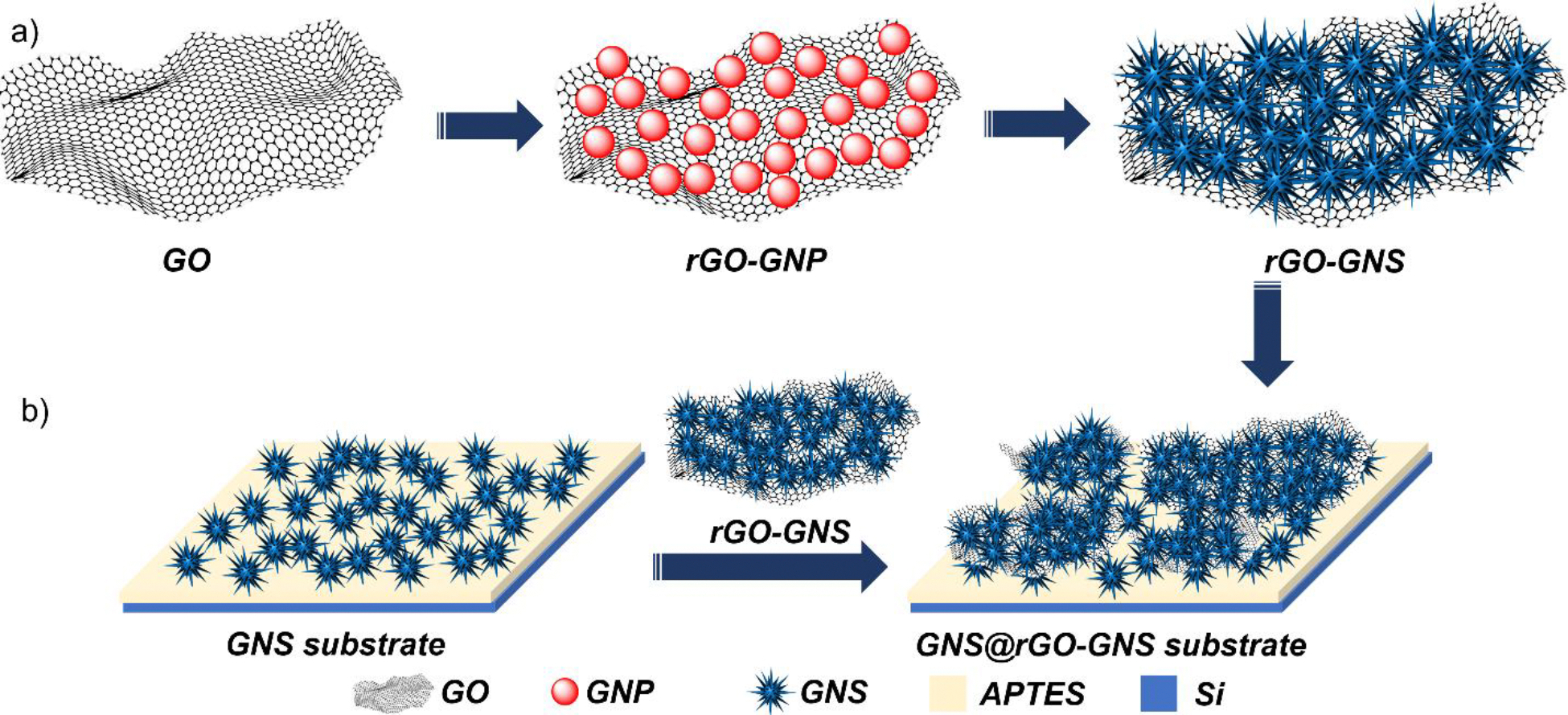
Schematic illustration of the GNS@rGO-GNS substrate preparation which involves fabrication of GNS onto the rGO nanosheets (rGO-GNS) (a), which is followed by the decoration of rGO-GNS on the GNS substrate (GNS@rGO-GNS substrate) (b).

**Figure 2.**
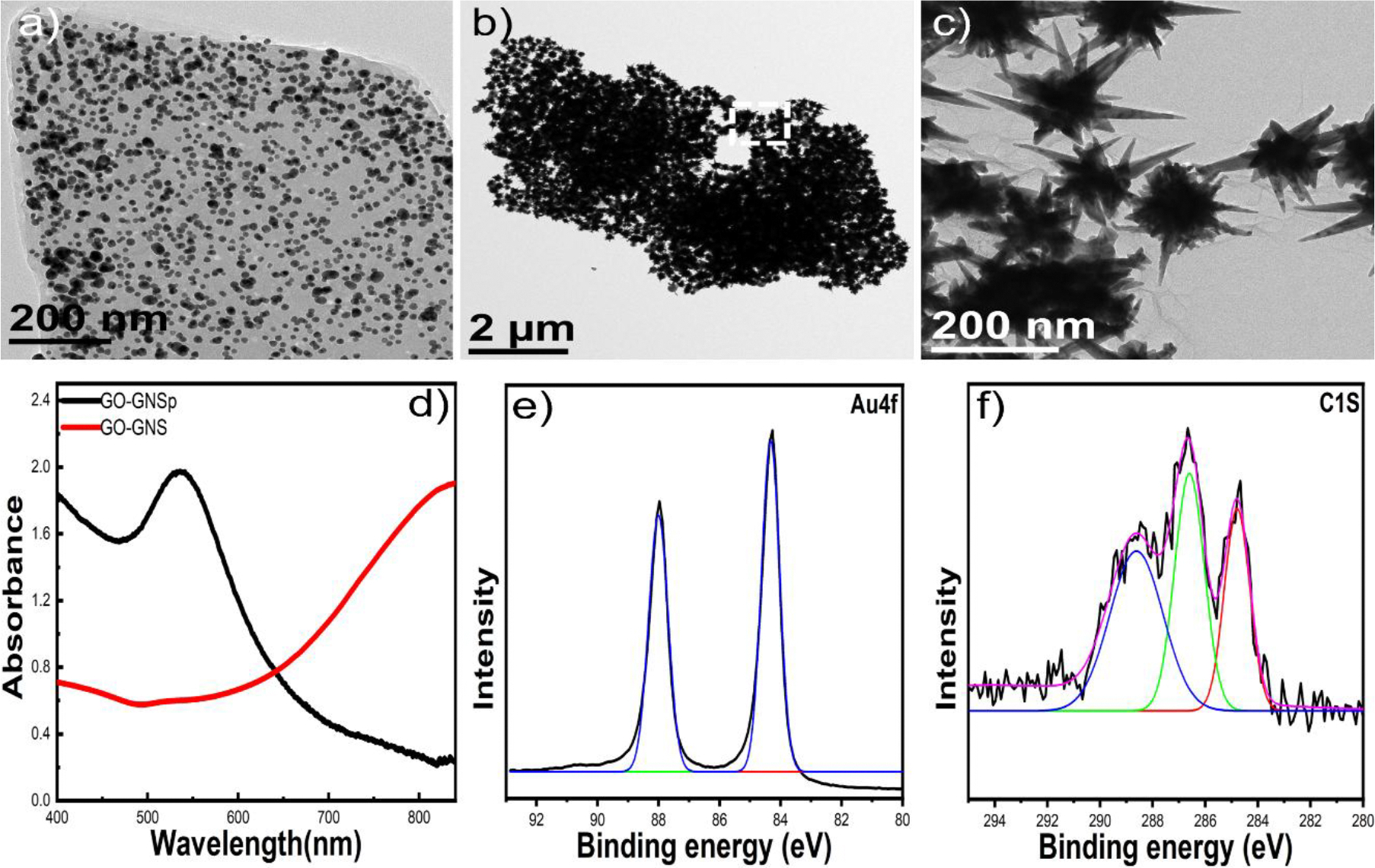
TEM image of rGO-GNSp (a) and rGO-GNS at different magnification (b-c). The UV-Vis absorbance spectra of rGO-GNSp and rGO-GNS (d). XPS spectra of rGO-GNS for Au 4f and C 1s indicating that GNS was decorated on rGO (e-f).

Schematic drawing of the synthesis process of the substrate GNS@rGO-GNS was shown in Figure 1. It is noteworthy that rGO plays a critical role in both the nucleation and growth of gold nanoparticles. The oxygen-containing functional groups on rGO introduce an overall negative surface charge, promoting the electrostatic attachment of Au (III), followed by its reduction and subsequent nucleation of gold nanoparticles^54^. Additionally, these oxygen-containing groups further facilitate the stable attachment of gold nanoparticles to the graphene surface. Once gold nanoparticles are formed and strongly anchored to the rGO sheet, they serve as nucleation centers for nanostar formation. Upon the addition of optimal concentrations of gold salts, ascorbic acid, and AgNO_3_ to the rGO-gold nanoparticle solution, the GNS nanostructures grow on these pre-attached gold nanoparticles, resulting in the formation of the desired morphology. In the first step, rGO-gold nanospheres (rGO-GNSp) were synthesized. As presented in Figure 2a, rGO exhibits a thin layer structure with gold nanospheres indicating the successful gold seeds incorporation on the 2D layered GO surfaces. In the second step, sharp spiked rGO-GNS was synthesized. As presented in Figure 2b-c, the GNSp were successfully transformed into GNS morphology. We have further characterized the rGO-GNS by UV-Vis and XPS. The UV-Vis absorbance spectra showed that the maximum plasmon resonance of GNSp was at 535 nm and it was red shifted to around 840 nm indicating that GNS morphology was formed (Figure 2d). Figure 2e-f showed the XPS spectra of Au 4f and C 1s of the rGO-GNS. Figure 2e exhibited the binding energy of Au 4f, where the Au 4f _7/2_ peak appeared at a binding energy of 84.3 eV and the Au 4f _5/2_ peak appeared at 87.9 eV, indicating the binding of GNS. Figure 2f exhibited the binding energy of C 1s, where the characteristic peaks of rGO were found at 284.5 eV (C=C), 286.7 eV (C-O) and 288.6 eV (O-C=O).

**Figure 3.**
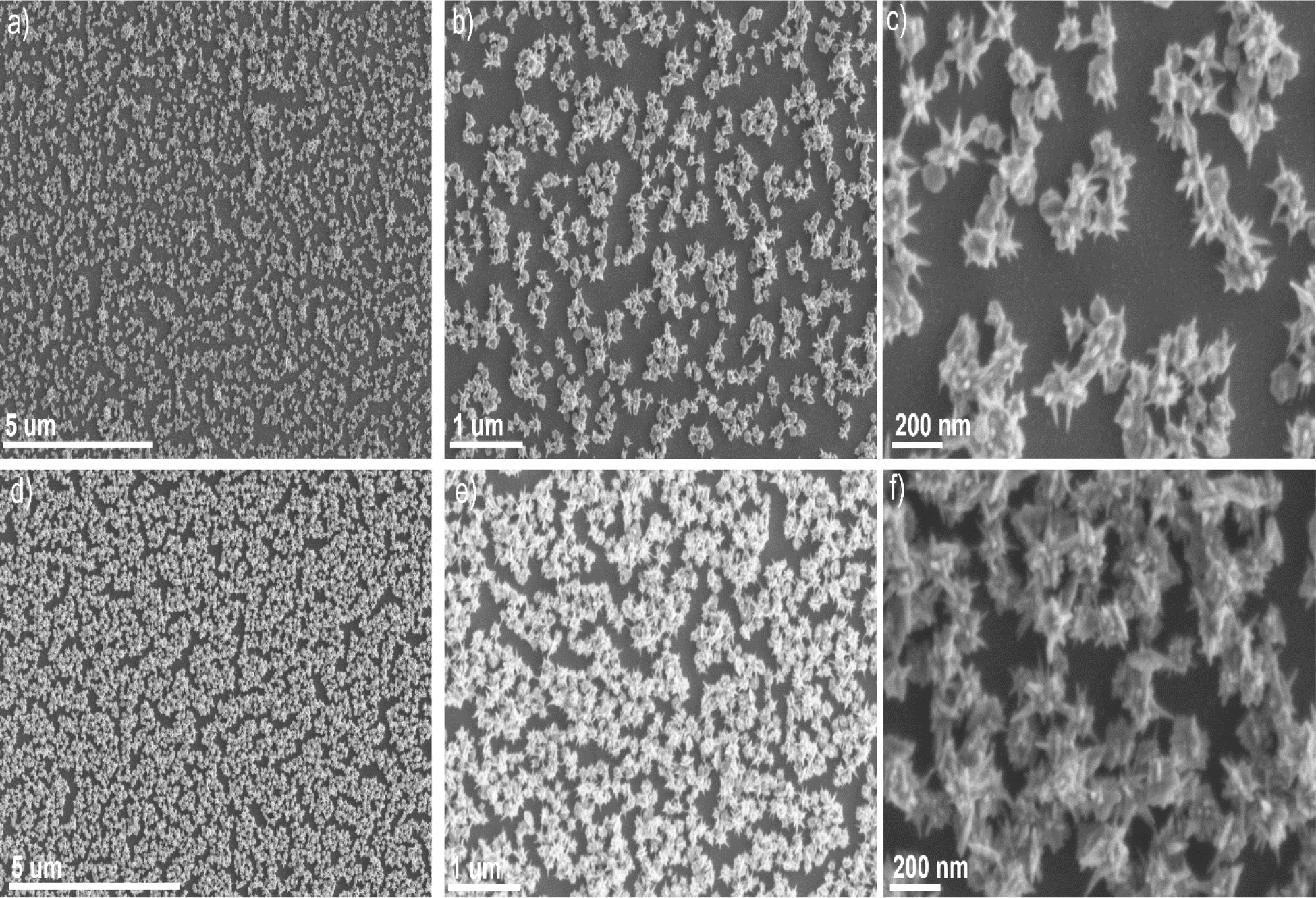
SEM images of GNS (a-c) and GNS@rGO-GNS (d-f) substrate at different magnification indicating that the substrates were highly monodispersed.

In the third step, rGO-GNS nanocomposite was decorated on a uniform GNS substrate. We prepared the GNS substrate by using the surfactant-free GNS. Figure 3a-c showed the SEM images of the GNS substrate at different magnification indicating that sharp spiked GNS were deposited on the APTES functionalized silicon wafer substrate. To achieve a highly monodispersed rGO-GNS on the GNS substrate, we incubated the GNS substrate for a short period of time (15 minutes). Figure 3d-f exhibited that the number of GNSs increased, indicating the successful loading of rGO-GNS on GNS surfaces. Furthermore, we observed aggregation of rGO-GNS and uneven patch formation for a long period of time incubation. The low-magnification TEM image in Figure 2b shows a dense arrangement of GNS, suggesting a possibility for aggregation. However, the high-magnification TEM image in Figure 2c reveals that while the GNS are closely positioned, they are not aggregated. Furthermore, the images in Figure 3d-f clearly demonstrate that the GNS are well-distributed and densely concentrated across the surface. This uniform distribution is important for achieving enhanced SERS performance, as it ensures a higher density of active sites for signal amplification. Figure S1 shows that uneven patch formation on GNS substrate after 30 minutes incubation time. We have utilized the GNS@rGO-GNS substrate for 15 minutes incubation time for further SERS study.

### SERS Performance of the Hybrid GNS@rGO-GNS Nanocomposite

To evaluate the SERS performances of the GNS@rGO-GNS substrate, we have analyzed thiophenol (TP) as model probe molecule using an excitation wavelength of 785 nm. In this study, we utilized a custom-built portable 785-nm laser for excitation. The use of different excitation lasers is beyond the scope of the current study and will be addressed in future research. It is important to note that SERS enhancement is not solely dependent on the excitation wavelength but is influenced by the morphology of the gold nanoparticles. For example, GNS exhibits superior SERS enhancement capabilities compared to other nanoparticle morphologies, such as gold nanorods and gold nanospheres. Furthermore, the SERS enhancement is influenced by the morphology of the GNS. For instance, our recent study demonstrated that SERS enhancement improves with increased spike sharpness and longer spike lengths in GNS structures^55^.

**Figure 4.**
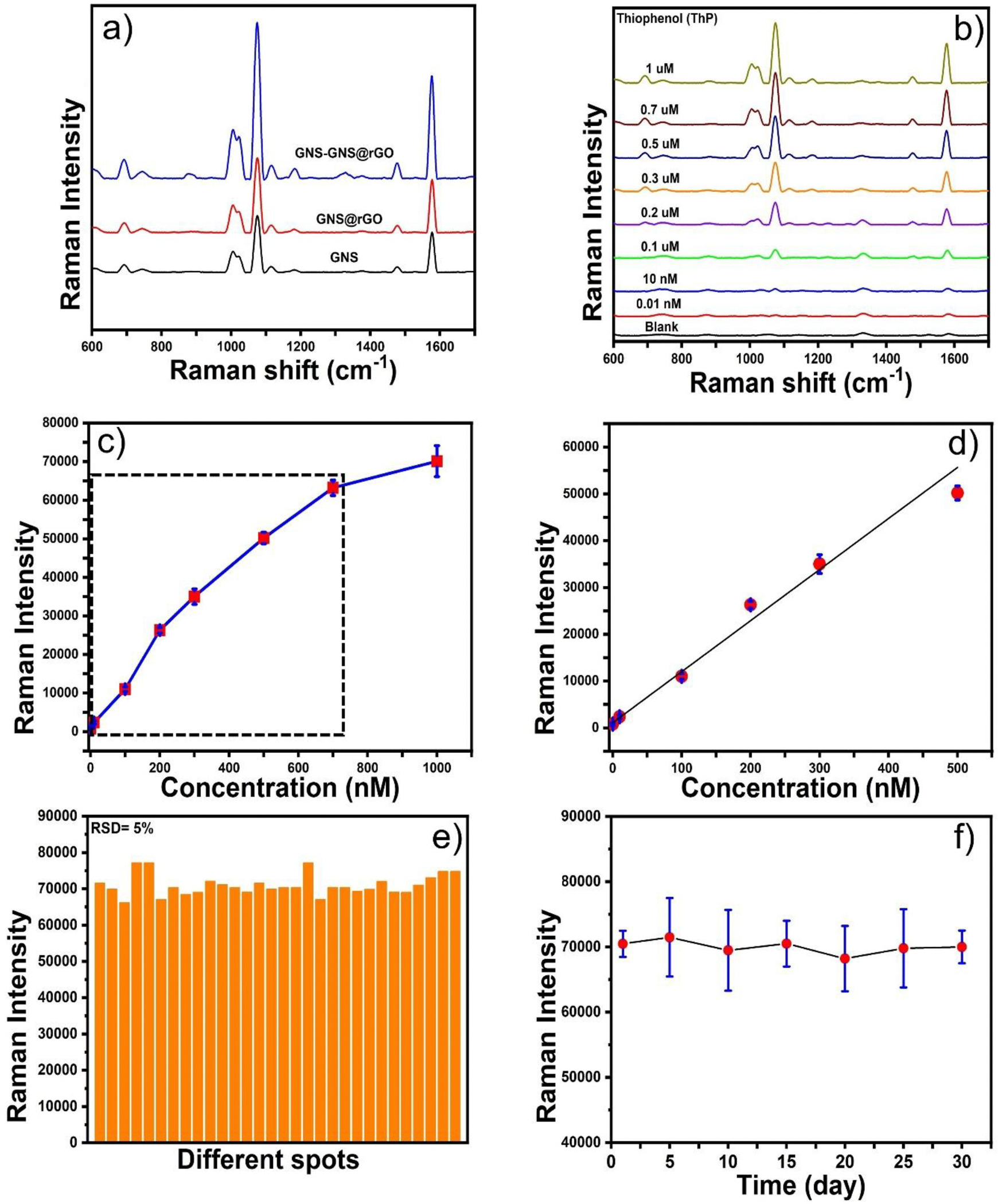
SERS comparison of GNS, GNS@rGO and GNS@rGO-GNS substrate (a). SERS spectra and calibration curve of TP with GNS@rGO-GNS substrate (b-d). SERS reproducibility and stability of the GNS@GO-GNS substrate (e-f).

We have compared the SERS enhancement of GNS, GNS@rGO, and GNS@rGO-GNS substrates. Figure 4a presents the SERS spectra of TP on different substrates, demonstrating that the SERS signal at 1075 cm^−1^ is more intense on the GNS@rGO substrate compared to GNS alone. Furthermore, the SERS intensity on the GNS@rGO-GNS substrate is more than twice that of both the GNS and GNS@rGO substrates. Interestingly, in the absence of GNS, no noticeable Raman signals of TP were observed. While in presence of GNS decorated on rGO, the distinct Raman spectra of TP for C-S stretching (1075 cm^−1^) and in-plane bending (1590 cm^−1^) vibrations appeared (Figure 4a). Moreover, the enhancement reached the maximum value for the GNS@rGO-GNS substrate. Generally, GNS possesses a higher number of inherent ‘hotspots’; thereby, the SERS enhancement efficiency of the hybrid nanocomposite GNS@rGO-GNS is better than that of GNS@rGO or GNS substrate. We have compared the SERS enhancement between the GNS and GNS@rGO-GNS substrates, showing that the SERS signal at 1075 cm^−1^ is more than twice as intense for the GNS@rGO-GNS substrate compared to the GNS substrate. As expected, the SERS enhancement of the GNS@rGO-GNS substrate was greater than that of the GNS substrate. This is likely due to the presence of GNS, which can generate a higher number of inherent “hotspots”, resulting in significant increase in the overall SERS signal. Additionally, the rGO nanostructure facilitates multiple π-π interactions between the aromatic rings of graphene or rGO and the aromatic analyte molecules, enhancing the charge transfer process and contributing to the chemical enhancement of the Raman signals. Therefore, the GNS@rGO-GNS substrate exhibits superior SERS enhancement compared to the GNS substrate.

Figure 4b exhibited the SERS spectra of TP at a concentration range from 1 μM to 0.01 nM. The highest SERS signal peak at 1075 cm^−1^ was chosen to establish the calibration curve of TP using GNS@rGO-GNS as the substrate. Figure 4c exhibited the calibration curve of TP, where the SERS signal intensity at 1075 cm^−1^ was plotted versus the concentration of TP. Moreover, Figure 4d displayed a linear relationship between the SERS intensity at 1075 cm^−1^ versus the concentration of TP in the concentration range from 0.3 nM to 10 pM. The linear equation is y = 109.11x + 1082.2, where y represents the Raman intensity of TP, and x denotes the concentration of TP. According to the calibration curve, the R^2^ value was 0.98. The LOD of TP was calculated to be 50 pM with a good signal-to-noise ratio (S/N= 3.5).

To quantify the results of uniformity of the SERS signal, we collected the SERS spectra of TP at 30 random points on the same substrate. Figure 4e shows the SERS intensity of TP at 1080 cm^−1^, where the relative standard deviation (RSD) for the 1080 cm^−1^ vibration was calculated to 5 %, indicating good uniformity and reproducibility of the prepared hybrid heterostructure substrate. To test the long-term stability of the GNS@rGO-GNS, we recorded the SERS spectra of TP for 30 days (Figure 4f). The long-term stability of the GNS@rGO-GNS may be attributed to the protection of sharp spikes of GNS by rGO layers. Overall, our SERS substrate shows superior enhancement performance and long-term stability that can meet the requirement for routine SERS measurements. We also examined the reusability of the substrate. However, our findings suggest that the substrate is not suitable for reuse. As shown in Figure S2, residual SERS signals persist even after washing. This result is expected, as the rGO in the substrate strongly interacts with analytes containing aromatic carbons.

### SERS Detection of Thiram by GNS@GO-GNS Nanocomposite

**Figure 5.**
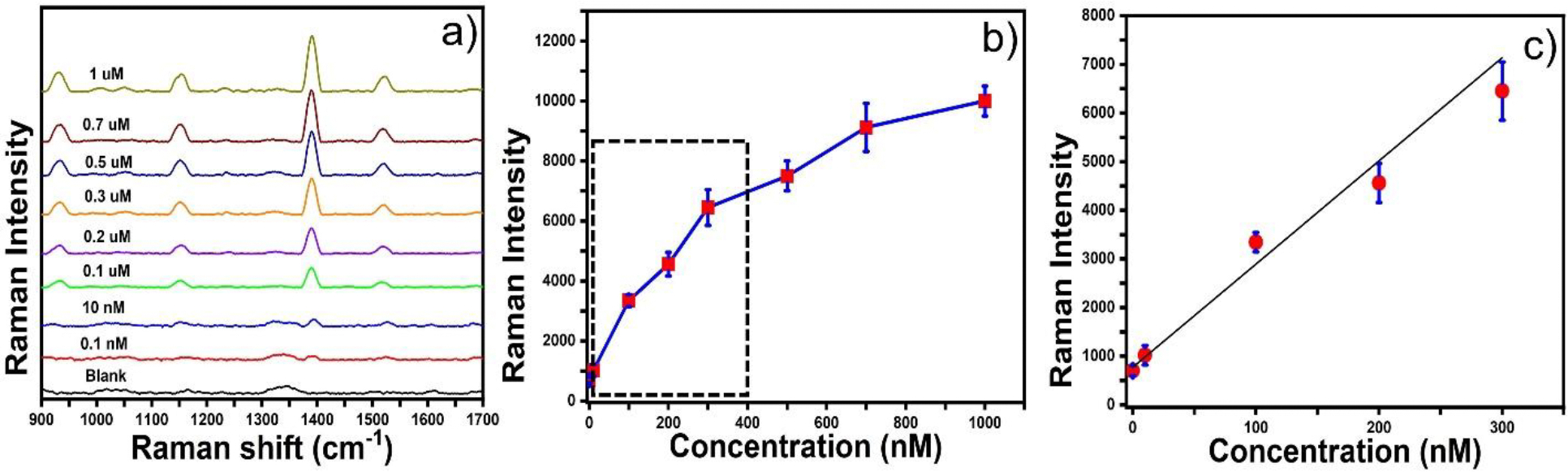
The SERS spectra and calibration curve of thiram (a-c).

Pesticides are generally used to prevent crop diseases and impede pests. It has been estimated by the world health organization (WHO) in the year 1985 that less than 0.1% of the pesticides were applied to crops^56^. However, excessive use of synthetic pesticides such as thiram in crop cultivation contaminates crops and soil, which causes serious ailments to human health and the environment. For instance, thiram can induce dyspnea, hyperactivity, convulsions, and severe fetal malformations^57^. Therefore, it is crucial to develop a rapid point-of-need detection platform for regulating and monitoring the levels of pesticides for food safety and environmental monitoring. The previously presented results demonstrate that the substrate exhibits excellent SERS sensitivity, high reproducibility, and long-term stability. Based on the promising results, we further utilized the GNS@rGO-GNS substrate for SERS-sensing of thiram in a real-life sensing scenario: river water. Figure 5a shows the SERS signal intensities of thiram at a concentration range from 1 uM to 0.1 nM. The SERS intensity of thiram at 1380 cm^−1^ was decreased by decreasing the concentration of thiram from 1 uM to 0.1 nM. Figure 5b displayed the calibration curve of the thiram, where the SERS signal intensity at 1380 cm^−1^ was plotted versus the concentration of thiram. Moreover, Figure 5c displayed a linear relationship between the SERS intensity at 1380 cm^−1^ versus the concentration of thiram in the concentration range from 0.3 μM to 0.1 nM. The linear equation is y = 21.2x + 772.35, where y represents the Raman intensity of thiram, and x denotes the concentration of thiram. According to the calibration curve, the R^2^ value was 0.98. The LOD of thiram was calculated to 50 pM with a good signal-to-noise ratio (S/N= 3.5). As the LOD of thiram by our SERS platform: GNS@rGO-GNS was 50 pM, it meets the detection standard’s requirement of the WHO, thus revealing excellent SERS performance indicating the potential application of GNS@GO-GNS in food safety assessments.

Several studies have reported the SERS detection of thiram using GO-modified plasmonic nanoparticle assemblies^58, 59^. For example, Zhu et al. developed a reduced graphene oxide-wrapped silver nanocube SERS substrate, achieving a limit of detection (LOD) of 44 nM for thiram^60^. Similarly, Chakraborty et al. reported a SERS substrate composed of silver nanoparticles decorated with reduced graphene, achieving an LOD as low as 10^−10^ M^58^. In our study, we achieved an LOD of 50 pM for thiram, demonstrating significantly higher sensitivity compared to previously reported GO-plasmonic nanoparticle assemblies for SERS-based thiram detection. Overall, we present the design of a nanoparticle substrate that integrates GNS with rGO, harnessing the unique properties of graphene alongside the chemical signal amplification and electromagnetic enhancement generated by the hot spots on the sharp spikes of GNS plasmonic nanoparticles. This synergy enables both high sensitivity and molecular specificity for thiram pesticide detection, providing a promising platform for sensitive SERS-based pesticide analysis.

## CONCLUSION

In summary, we have demonstrated a plasmonic hybrid heterostructure system: GNS@rGO-GNS by combining the electromagnetic enhancement properties of GNS and the chemical enhancement properties of rGO. After comparing SERS enhancing performance of the substrates including GNS, GNS@rGO, and GNS@rGO-GNS by using TP as a SERS reporter molecule, we observed that the combination of GNS, and rGO-GNS nanocomposites had the highest SERS enhancements, which could be attributed to the generation a higher number of inherent “hotspots” formed between the GNS and rGO-GNS and the additional chemical enhancement induced by rGO. The optimized hybrid heterostructure system was used to detect thiram, achieving an LOD of 50 pM. It is worth mentioning that our SERS platform can detect thiram, which meets the WHO’s requirement for the maximum limit of thiram, indicating good sensitivity and it can be applied for environmental monitoring assessments.

## Supplementary Material

Supporting Information
