# Supplementary material for: Plasmonic Hybrid Heterostructure Based on Reduced Graphene Oxide-Gold Nanostars Composite for Sensitive SERS Sensing
Source: Appl Spectrosc. Author manuscript; Available in PMC 2026 Jan 6. (PMC12768889; doi:10.1177/00037028251344628)
Supplement: Supporting Information [file NIHMS2126897-supplement-Supporting_Information.docx]

**Plasmonic Hybrid Heterostructure Based on Reduced Graphene Oxide-Gold Nanostars Composite for Sensitive SERS Sensing**

*Supriya Atta,* ^a, b^ *Tamer Sharaf,* ^a, b, c^ *Tuan Vo-Dinh* ^a,^ ^b, d‡^

*^a^* Fitzpatrick Institute for Photonics, *^b^* Department of Biomedical Engineering, *^c^* Department of Physics, Ain Shams University, Cairo, Egypt, *^d^* Department of Chemistry, Duke University, Durham, NC 27708, USA.


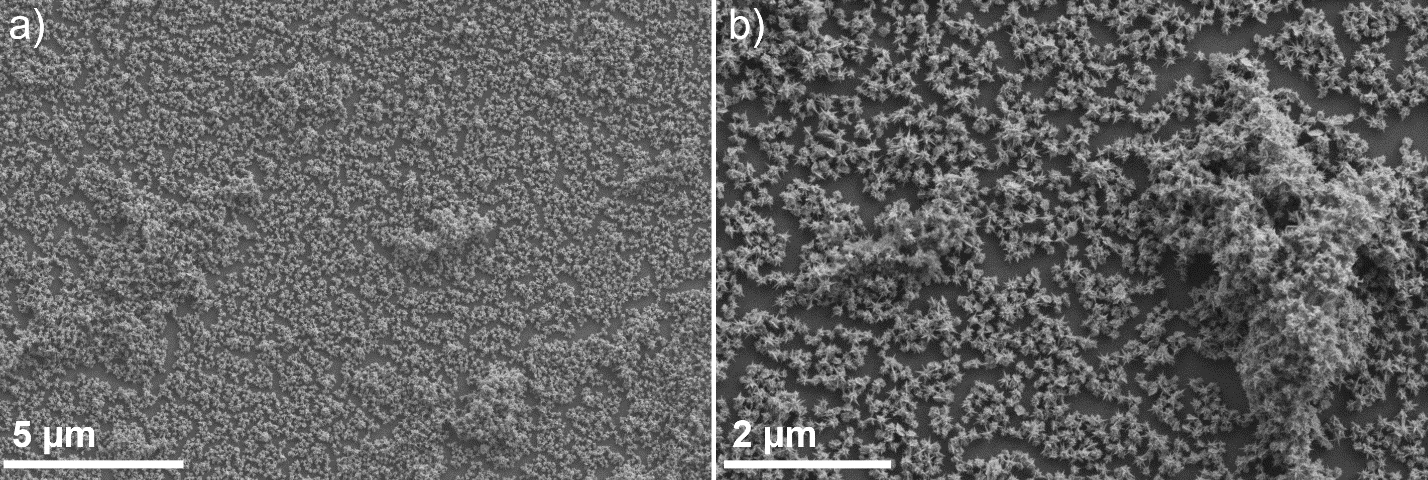


**Figure S1.** SEM images of the GNS@GO-GNS substrate after 30 minutes incubation time.


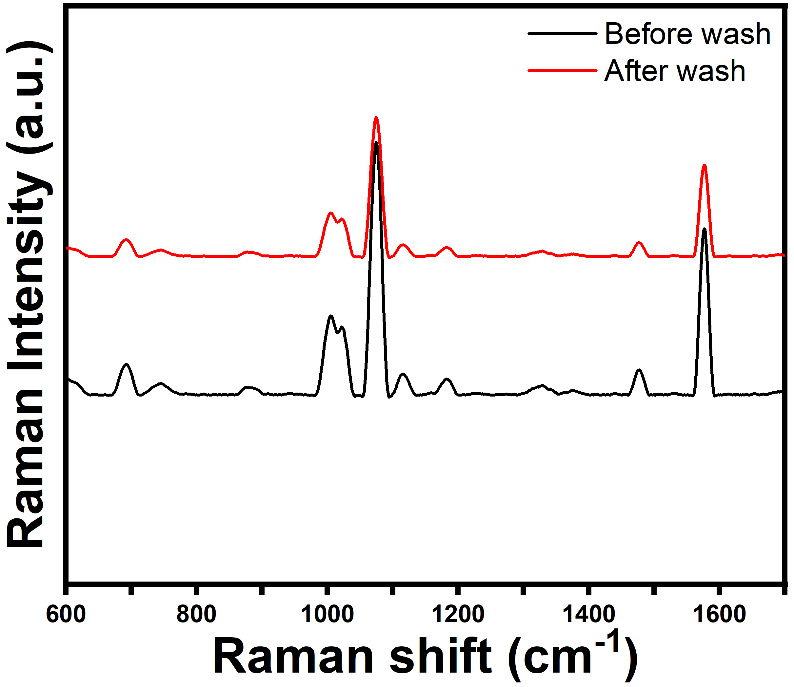


**Figure S2.** SERS spectra of TP before and after washing.
